# Lymph node surgery after centralization of penile cancer care in Sweden: Extent of use and complications

**DOI:** 10.1002/bco2.70220

**Published:** 2026-04-30

**Authors:** Åsa Warnolf, Gediminas Baseckas, Dominik Glombik, Oskar Hagberg, Peter Kirrander, Kimia Kohestani, Per Nordlund, Erik Persson, Fredrik Liedberg, Axel Gerdtsson

**Affiliations:** ^1^ Department of Translational Medicine Lund University Malmö Sweden; ^2^ Department of Urology Skåne University Hospital Malmö Sweden; ^3^ Department of Urology, Faculty of Medicine and Health Örebro University Örebro Sweden; ^4^ Department of Urology, Institute of Clinical Sciences, Sahlgrenska Academy University of Gothenburg Gothenburg Sweden; ^5^ Department of Urology, Sahlgrenska University Hospital Region Västra Götaland Gothenburg Sweden; ^6^ Department of Urology Södersjukhuset Stockholm Sweden; ^7^ Regional Cancer Center Uppsala Örebro Uppsala Sweden

**Keywords:** centralization, complications, lymph node surgery, penile cancer

## Abstract

**Objectives:**

The objective of this study is to compare the use of lymph node surgery and complication rates before and after centralization of penile cancer (PeCa) surgery in Sweden. In January 2015, curative surgical care for PeCa was centralized to the Skåne University Hospital in Malmö and the Örebro University Hospital in Örebro.

**Patients and Methods:**

All 1079 patients with invasive PeCa in the Swedish National Penile Cancer Register (NPECR) diagnosed between 2009 and 2020 were included, 458 before and 621 after centralization. The proportion of patients subjected to lymph node surgery 2009–2014 versus 2015–2020 was compared using Pearson's Chi‐squared test. Odds ratios (ORs) for complications were calculated using a logistic regression model adjusting for age, nodal stage and extent of penile surgery. Continuous variables were presented as medians and compared using Wilcoxon's rank sum test.

**Results:**

Before centralization, 270/458 (59%) of patients were subjected to lymph node surgery compared to 474/621 (76%) after 2014. Overall complication rate for patients undergoing such surgery was unaltered before and after centralization. The incidence of any complications increased after Dynamic Sentinel Lymph Node Biopsy from 18% (22/122) to 36% (91/256), corresponding to an adjusted OR of 1.89 (95% confidence interval [CI] 1.09–3.27). For lymphocele, lymphedema or infection after modified or radical inguinal or pelvic lymphadenectomy, the adjusted OR was 0.47 (95% CI 0.29–0.75) after centralization. The register‐based setting is a study limitation.

**Conclusions:**

The proportion of men undergoing lymph node surgery increased, and the risk of lymphocele, lymphedema and infection after inguinal and pelvic lymph node surgery decreased after centralization of PeCa care.

## 1 INTRODUCTION

The incidence of penile cancer (PeCa) in Sweden is 2/100 000 men.^1^ Tumour dissemination primarily occurs through the inguinal lymph nodes, and the presence of lymph node metastasis is the most important prognostic factor for survival.^2^ At diagnosis, up to 22% of clinically node negative patients are found to have micro‐metastases after surgical lymph node staging.^3^ The standard method for inguinal lymph node staging in clinically node negative patients is dynamic sentinel lymph node biopsy (DSNB), which reduces morbidity compared to modified inguinal lymphadenectomy (MIL).^4^ Patients with confirmed inguinal lymph node metastases are treated with radical inguinal lymphadenectomy (RIL). Compared to MIL, DSNB preceded by preoperative ultrasound, and fine needle aspiration offers higher diagnostic accuracy and a lower complication rate.^5^, ^6^

A weekly national multidisciplinary team conference (MDT) for penile cancer where all new PeCa patients are discussed was launched in 2013 in Sweden, and 2 years later, in January 2015, curative surgical care for penile cancer was centralized to Skåne University hospital in Malmö and Örebro University hospital in Örebro.

This study aims to investigate whether the use of lymph node surgery increased after centralization of PeCa care in Sweden and if complications after such surgery decreased.

## 2 PATIENTS AND METHODS

**FIGURE 1:**
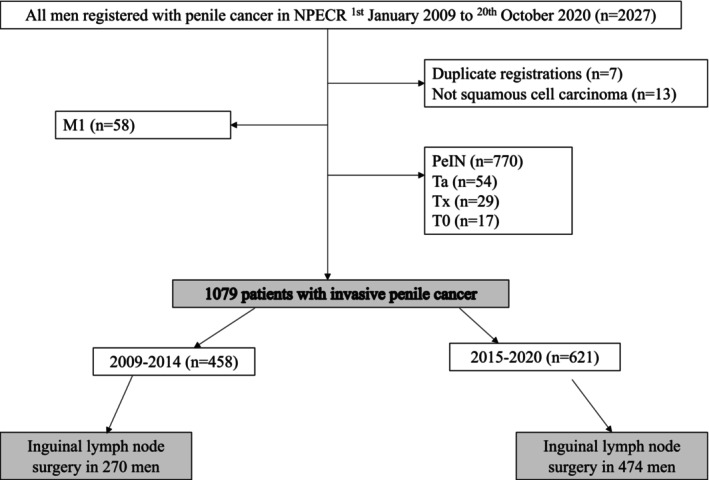
Consort diagram.

All 2027 men diagnosed with PeCa between 1 January 2009 and 20 October 2020 registered in the National Penile Cancer Register (NPECR) were extracted. The NPECR is a population‐based register with high completeness compared to the mandatory Swedish cancer register.^7^ After exclusions due to multiple registrations, other histologies than squamous cell carcinoma, presence of distant metastases at diagnosis and noninvasive PeCa (PeIN and Ta), 1079 patients with invasive PeCa remained (Figure 1). In 79 men registered as cTx, instead, a cT‐stage was derived from their corresponding pT stage given an invasive pT stage (i.e., stage pT1 or higher). In total, 744 men were subjected to lymph node surgery, either by DSNB, MIL, RIL or pelvic lymph node dissection (PLND) for whom analyses of complications were performed stratifying lymph node surgery as DSNB versus MIL/RIL/PLND. If the individual was subjected to more than one type of lymph node surgery, they were stratified according to the most extensive procedure. In 13 men receiving lymph node surgery but without information about type or extent, the type of lymph node surgery was categorized in the MIL/RIL/PLND group.

The variables extracted from the NPECR were age, clinical stage (cT), clinical nodal stage (cN), type of penile surgery (local resection or glansectomy, partial amputation or total amputation), treatment intent (curative/palliative), chemotherapy (neoadjuvant/adjuvant/palliative), pathological stage (pT), pathological nodal stage (pN) and grade.

When the 7th edition of TNM for penile cancer in 2010 was published, there was an update regarding both T and N stages,^8^ where Stage T1 was substratified into T1a and T1b. This update was not incorporated in the NPECR until 1 September 2015; therefore, all T1 stages (i.e., cT1, cT1a and cT1b) were aggregated as cT1 in this study. At the same time, nodal stage definitions were altered so that N1 included up to two unilateral inguinal metastases, instead of previously one. However, data in NPECR were not available to retrospectively update nodal stage according to this change, and therefore, nodal stage was categorized according to the primary registration. Subsequently, in the 8th TNM edition, the definition of Stages T2 and T3 changed to invasion of corpus spongiosum and corpus cavernosum, respectively, and urethral invasion no longer played a role in staging. Consequently, patients with Stages T2 and T3 had to be categorized according to their primary registration in NPECR.

Complications within 90 days of surgery were also retrieved from NPECR. Complications registered in NPECR are reoperation, bleeding, infection, skin necrosis, meatal stenosis, lymphocele, thrombosis, lymph edema or other. To associate complications more directly related to lymph node surgery, separate analyses for a subset of complications including lymphocele, lymphoedema and infections were also investigated.

### 2.1 Statistical analysis

The participants were stratified according to date of diagnosis, that is, before and after centralization of penile cancer surgery in Sweden 1 January 2015 (2009–2014 vs. 2015–2020). Proportions were presented as percentages, and descriptive statistics were applied for continuous variables with medians and Inter Quartile Ranges (IQR). Pearson's Chi‐squared test compared the proportion of patients receiving lymph node surgery. To explore temporal trends before centralization, a logistic regression analysis 2009–2014 was performed. A Wilcoxon's rank sum test was applied when comparing median age. Odds ratios (ORs) with 95% confidence intervals (CIs) for complications after surgery for the two time periods were calculated using a multi‐variable logistic regression model adjusting for age, nodal stage and type of surgery for the penile primary tumour. As a sensitivity analysis, all analyses were also performed without the 13 men where the extent of lymph node surgery was not known. For all statistical analysis, the R statistical package Version 4.1.1 was used.

The study was approved by the research ethics board at Gothenburg University, Sweden (EPN 2022‐04075‐01).

## 3 RESULTS

In total, 1079 men with invasive PeCa (Stage T1 and above) were registered in the NPECR between 1 January 2009 and 20 October 2020 (Figure 1). Before centralization of curative penile cancer surgery in Sweden, 458 men were diagnosed 2009–2014 and, subsequently, after centralization, 621 men were diagnosed 2015–2020. Individual patient characteristics for both time periods and the total population are presented in Table 1. Median age was 70 years (IQR 62–79) before centralization and 71 years (IQR 64–78) after centralization (*p* = 0.5).

**TABLE 1:** Patient characteristics.

Year of diagnosis	2009–2014	2015–2020
Number of patients (%)	458 (42)	621 (58)
Median age at diagnosis (IQR)	70 (62–79)	71 (64–78)
cT stage		
cT1	210 (46)	322 (52)
cT2	161 (35)	187 (30)
cT3	78 (17)	105 (17)
cT4	9 (2)	7 (1)
cN stage		
N0	214 (47)	404 (65)
N1	48 (10)	57 (9)
N2	33 (7)	27 (4)
N3	15 (3)	16 (3)
Nx	148 (32)	117 (19)
M stage		
M0	377 (82)	582 (94)
MX	81 (18)	39 (6)
Type of penile surgery^a^		
Glansectomy or less	176 (40)	272 (48)
Partial amputation	186 (42)	184 (32)
Total amputation	77 (18)	113 (20)
Type of lymph node surgery		
None	188 (41)	147 (24)
DSNB	126 (28)	288 (46)
RIL/MIL	88 (19)	127 (20)
PLND	56 (12)	59 (10)
Treatment intent^b^		
Curative	412 (96)	562 (94)
Palliative	17 (4)	36 (6)
Chemotherapy^c^		
No	409 (92)	542 (90)
Yes, neoadjuvant	0 (0)	42 (7)
Yes, adjuvant	3 (1)	10 (2)
Yes, palliative	1 (0)	3 (0)
Yes, unknown type	33 (7)	2 (0)
pT stage		
pT0, pTis and pTa	15 (3)	31 (5)
pT1	160 (35)	231 (37)
pT2	164 (36)	209 (34)
pT3	82 (18)	117 (19)
pT4	5 (1)	3 (0)
pTX	32 (7)	30 (5)
pN stage		
pN0	162 (60)	325 (69)
pN1	35 (13)	64 (14)
pN2	37 (14)	27 (6)
pN3	22 (8)	48 (10)
pNx	14 (5)	10 (2)
Grade		
G1	109 (24)	116 (19)
G2	171 (37)	237 (38)
G3	95 (21)	199 (32)
GX	83 (18)	69 (11)
Complications^d^		
None	304 (73)	355 (67)
Yes	115 (27)	177 (33)

Abbreviations: DSNB, dynamic sentinel lymph node biopsy; IQR, interquartile range; MIL, modified inguinal lymphadenectomy; PLND, pelvic lymph node dissection; RIL, radical inguinal lymphadenectomy.

^a^ Missing data before and after centralization in 19 and 52 patients, respectively.

^b^ Missing data before and after centralization in 29 and 23 patients, respectively.

^c^ Missing data before and after centralization in 12 and 22 patients, respectively.

^d^ Missing data before and after centralization in 39 and 89 patients, respectively.

Among the 1079 men, 744 men (69%) were subjected to DSNB or lymph node surgery (MIL/RIL/PLND) during the whole study period. The proportion of patients subjected to any lymph node surgery before centralization was 270/458 (59%) (2009–2014) compared to 474/621 (76%) (*p* < 0.001) after (Tables 1 and S1). Among men with pT1 stage, lymph node surgery rate increased from 50% before 2015 to 71% after, and for pT2 stages and above, the proportions were 73% before and 88% after centralization, respectively. Median age among the 744 men subjected to lymph node surgery was 67 (IQR 60–73) years before centralization compared to 70 (IQR 62–76) years after (*p* = 0.001). Patient characteristics for men with PeCa subjected to lymph node surgery 2009–2020 are given in Table 2. Tables 1 and 2 show that the patient population remained largely unchanged before and after centralization in terms of age, nodal stage and tumour characteristics. The proportion of patients not undergoing lymph node dissection decreased from 41% before 2015 to 24%, reflecting a change in treatment patterns after centralization. In total, only 16 men with cN2/N3 did not receive any lymph node surgery, of whom 10 were treated before 2015. National MDTs that were introduced 2 years prior may have influenced surgical treatment already then, even if no increased temporal use of lymph node surgery was observed before 2015 (Table S1, *p* = 0.053). Before centralization, 42% (113/266) of lymph node surgeries were performed in Malmö or Örebro. Complication rate in Malmö/Örebro was 35% (39/111) pre‐centralization compared to 31% (48/153) at other centres. Distribution of centre for lymph node surgery pre‐centralization and corresponding complications (lymphedema/lymphocele/infection) is shown in Figure S1.

**TABLE 2:** Patients receiving lymph node surgery.

	DSNB		MIL/RIL/PLND	
Year of diagnosis	2009–2014	2015–2020	2009–2014	2015–2020
Number of patients (%)	126	288	144	186
Age at diagnosis				
Median age at diagnosis (IQR)	66 (62–74)	71 (63–76)	67 (60–72)	69 (60–75)
cT stage				
cT1	57 (45)	152 (53)	45 (31)	68 (37)
cT2	49 (39)	96 (33)	62 (43)	66 (35)
cT3	18 (14)	40 (14)	36 (25)	49 (26)
cT4	2 (2)	0 (0)	1 (1)	3 (2)
cN stage				
N0	83 (66)	230 (80)	49 (34)	81 (44)
N1	4 (3)	12 (4)	32 (22)	43 (23)
N2	1 (1)	2 (1)	26 (18)	22 (12)
N3	0 (0)	1 (0)	11 (8)	12 (6)
Nx	38 (30)	43 (15)	26 (18)	28 (15)
Type of penile surgery				
Glansectomy or less	56 (44)	143 (53)	39 (27)	56 (32)
Partial amputation	59 (47)	76 (28)	61 (42)	70 (40)
Total amputation	11 (9)	53 (19)	44 (31)	51 (29)
Missing	0	16	0	9
Treatment intent				
Curative	125 (100)	285 (100)	141 (99)	176 (95)
Palliative	0 (0)	0 (0)	1 (1)	9 (5)
Missing	1	3	2	1
Complications				
None	100 (82)	165 (64)	65 (47)	87 (53)
Any	22 (18)	91 (36)	73 (53)	77 (47)
Lymphedema/lymphocele/infection^a^	19 (16)	70 (27)	68 (49)	60 (37)
Missing	4	32	6	22

DSNB=Dynamic sentinel lymph node biopsy, MIL = Modified inguinal lymphadenectomy, RIL = Radical inguinal lymphadenectomy, PLND = Pelvic lymph node dissection.

^a^ Lymphedema/lymphocele/infection represents a subset of ‘any’ complications.

**FIGURE 2:**
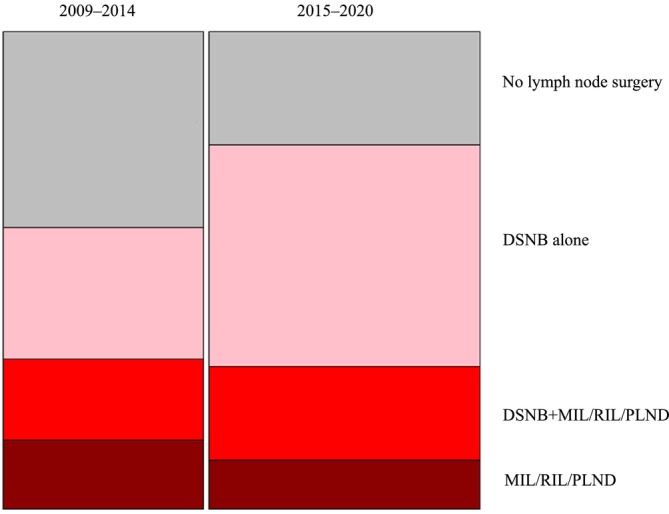
Proportion of patients receiving lymph node surgery before and after centralization. DSNB, dynamic sentinel lymph node biopsy; MIL, modified inguinal lymphadenectomy; RIL, radical inguinal lymphadenectomy; PLND, pelvic lymph node dissection.

Similarly, the proportion of clinically node‐negative patients undergoing lymph node surgery increased from 132/214 (62%) to 311/404 (77%) after centralization, and in the subgroup with cT1 stage, the proportions were 102/210 (49%) and 220/322 (68%) before and after centralization, respectively. The increased use of lymph node surgery was due to increased use of DSNB (Figure 2).

Clinical nodal stage distribution showed a difference before and after centralization, where the proportion cNx decreased from 148/458 (32%) to 117/621 (19%) after centralization, whereas pN‐stage distribution was equal over time. Similarly, the proportion Mx was higher before centralization than after, 81/458 (18%) versus 39/621 (6%), respectively.

For patients operated with lymph node surgery, the overall complication rate for those receiving lymph node surgery was similar before and after centralization, 94/256 (36%) and 168/420 (40%), respectively (*p* = 0.4). In the subset of patients operated with DSNB, the complication rate was 18% (22/122) before and 36% (91/256) after centralization. For patients undergoing more extensive lymph node surgery with MIL/RIL/PLND, the corresponding proportions were 53% (73/138) and 47% (77/164), respectively.

Considering only complications with a more obvious causal relation to lymph node surgery per se (i.e., lymphocele, lymphedema and/or infection), complication rates after DSNB were 16% (19/122) before centralization and 27% (70/256) after. For patients subjected to any combination of MIL/RIL/PLND, the corresponding figures were 49% (68/138) and 37% (60/164), respectively. In a logistic regression model adjusting for age, extent of penile primary tumour surgery and N‐stage the OR was 0.47 (CI 0.29–0.75, *p* = 0.002) with the years 2009–2014 as reference in those patients operated with MIL/RIL/PLND (Table 3).

**TABLE 3:** Logistic regression model on complications after DSNB versus MIL/RIL/PLND.

		Number of patients	Number of events (%)	Odds ratio ^a^	CI95%	*p* value
All complications after DSNB	2009–2014	126	22 (17)	1	–	–
	2015–2020	288	91 (32)	1.89	1.09–3.27	0.023
Lymphedema, lymphocele or infection after DSNB	2009–2014	126	19 (15)	1	–	–
	2015–2020	288	70 (24)	1.67	0.93–2.99	0.087
All complications after MIL/RIL/PLND	2009–2014	144	73 (51)	1	–	–
	2015–2020	186	77 (41)	0.64	0.41–1.01	0.057
Lymphedema, lymphocele or infection after MIL/RIL/PLND	2009–2014	144	68 (47)	1	–	–
	2015–2020	186	60 (32)	0.47	0.29–0.75	0.002

Abbreviations: DSNB, dynamic sentinel lymph node biopsy; MIL, modified inguinal lymphadenectomy; PLND, pelvic lymph node dissection; RIL, radical inguinal lymphadenectomy.

^a^ Adjusted for age (<70; at least 70), nodal stage (N0/NX; N+) and extent of penile primary tumour surgery (glansectomy or less; partial penectomy; and total amputation).

In the sensitivity analysis excluding the 13 men, where extent of lymph node surgery was not known, the results remain unchanged (data not shown).

## 4 DISCUSSION

A higher proportion of men with invasive PeCa in Sweden underwent nodal surgery following centralization of PeCa care (Table S1). The proportions of cNx and cMx were decreased after centralization, suggesting more granular and standardized staging practice. As lymph node status is the most important prognostic factor for men with penile cancer, this is a positive development. Regarding complications, we observed a higher complication rate for patients undergoing DSNB after centralization, whereas the complication rate after MIL/RIL/PLND decreased from 49% to 37%, corresponding to an OR of 0.47. Thus, patients with more advanced disease appeared to benefit most from centralization in terms of reduced complication risk.

One possible explanation for the increased complication rate in patients subjected to DSNB after centralization could be that the median age for patients undergoing lymph node surgery was higher after centralization (67 years vs. 70 years, respectively). Assuming that older patients are more comorbid than younger, at least on a group level, referring more comorbid men to lymph node surgery after centralization might increase the risk of complications. Current practice to reduce complications includes antibiotic prophylaxis for all patients and the use of a drainage tube in those undergoing radical lymph node dissection. However, the register lacks information on these parameters and disentangling how and to what extent any of these factors contributed to the observed differences in complication rates was not possible. Another explanation for increased proportion complications after DSNB could be that the total number of healthcare employees involved in the care of PeCa decreased as a consequence of centralization, and with increased experience, a higher awareness and subsequent registration of complications occurs. On the other hand, the proportion of missing values for complications seemed to increase after centralization, which partly might be a consequence of changes in the NPECR reporting forms and the change from primarily using paper forms to online forms for reporting where the field ‘complications to surgical treatment’ is mandatory.

The time period chosen for this study was based upon the update in the NPECR in 2020, when the forms for reporting to the NPECR went through a major update after which complications were registered only for 30 days, instead of 90 days after surgery. Even if there was a notable difference in the percent of invasive penile cancer patients receiving lymph node surgery before and after centralization, the extent of lymph node surgery was probably increasing already before 2015 (Table S1), because the national MDT started already in 2013. For example, the introduction of a national MDT and implementation of the first national guidelines on PeCa in 2013 could have contributed already before the centralization although not significantly (Table S1).^9^

Another limitation is the register‐based setting of this study. Reclassification of patients according to the updated TNM was consequently not possible, which is a limitation. Furthermore, some individuals were missing, although the recently published validation study on the NPECR showed high coverage.^7^ On the other hand, the NPECR is, to our knowledge, the largest national penile cancer register in the world. There is an ongoing update of the research database PenCBaSe, which was created to facilitate research by linking the NPECR to other population‐based healthcare registers, demographic databases and Statistics Sweden.^10^ Neither overall survival nor cancer‐specific survival was available in this data set, but future studies in PenCBaSe will enable investigating whether the increased use of lymph node surgery is corresponding to improved survival outcomes, which has been previously suggested.^11^

A recently published article from a tertiary referral centre in Norway describes 201 consecutive patients regarding early and late complications.^12^ Their overall early complication rate was 45% even though the extent and use of lymph node surgery are comparable to the earlier years of this study (Table S1). In that study, the complication rate after penile surgery was reported separately. Because PeCa surgery in Sweden is performed in one session (especially DSNB) and not as a staged procedure, complications from both penile and lymph node surgery are reported. Thus, comparing complication rates between that study and the current one is not possible. A worldwide expert consensus created a classification system to report complications after inguinal lymph node dissection.^13^ The NPECR and other registers for penile cancer might in the future implement such improvements when registering complications, as this likely would improve the validity of the registration of complications after lymph node surgery in the register.

DSNB is recommended as standard of care in both Swedish and European guidelines for clinically node‐negative PeCa patients with high grade Stage T1 and higher T stages.^9^, ^14^ Despite this, publications show low adherence to this recommendation, and Mistretta et al.^15^ report that only 25% receive guideline‐recommended ILND in the United States. High complication rates are considered one reason for omitting nodal staging, despite guideline recommendations.

A consensus group against cancer stated in 2013 that MDTs are essential instruments for efficient cancer care.^16^ It is likely that the increased use of lymph node surgery observed in the current study was mediated through the national weekly MDT. In addition to MDTs, supra‐regional centres for penile cancer care are now highly recommended, which has now been implemented in many countries.^17^, ^18^, ^19^, ^20^ Such a transition of PeCa care to high volume centres is supported by the current study outcomes but also by an increased use of perioperative chemotherapy observed after the centralization.^21^ Consequently, for countries with a similar healthcare structure as Sweden, positive gains from centralization of PeCa care might be expected. In addition, hypothesis generating findings suggest both an increased use of organ‐sparing PeCa surgery and association with overall survival by centralizing PeCa care and increased hospital volume, respectively.^22^, ^23^

## 5 CONCLUSIONS

A higher proportion of men undergo recommended invasive lymph node staging and lymph node surgery after 2015 when centralization of curative PeCa surgery was implemented in Sweden. The increased complication rate after DSNB might be a consequence of better adherence to guideline recommendations, also for older and comorbid men. On the other hand, those men undergoing more extensive lymph node surgery seem to profit from the centralization by having a reduced risk of lymphocele, lymphedema and infection by approximately 50%.

## AUTHOR CONTRIBUTIONS

**Åsa Warnolf**: Conceptualization; funding acquisition; investigation; writing—original draft. **Gediminas Baseckas**: Investigation; writing—review and editing. **Dominik Glombik:** Investigation; writing—review and editing. **Oskar Hagberg:** Formal analysis; writing—review and editing. **Peter Kirrander:** Investigation; writing—review and editing. **Kimia Kohestani:** Investigation; writing—review and editing. **Per Nordlund:** Investigation; writing—review and editing. **Erik Persson:** Investigation; writing—review and editing. **Fredrik Liedberg:** Conceptualization; funding acquisition; investigation; writing—original draft. **Axel Gerdtsson:** Conceptualization; funding acquisition; investigation; writing—original draft.

## ACKNOWLEDGEMENTS

The authors have nothing to report.

## CONFLICT OF INTEREST STATEMENT

The authors declare no conflicts of interest.

## Supporting information


**Table S1.** Annual number of patients with invasive penile cancer and proportion receiving lymph node surgery over time, 2009–2020. No significantly increased temporal use of lymph node surgery was observed 2009–2014 (P = 0.053 from a univariate logistic regression with year as continuous covariate, restricted to the years before 2015).

**Figure S1.** Distribution of complication rates (lymphedema/lymphocele/infection) after lymph node surgery before 2015 for Malmö/Örebro vs all other centers.
