# Supplementary material for: Lymph node surgery after centralization of penile cancer care in Sweden: Extent of use and complications
Source: BJUI Compass. 2026 Apr 30;7(5):e70220. doi: 10.1002/bco2.70220 (PMC13133551; doi:10.1002/bco2.70220)
Supplement: Supplementary file 1 — Table S1. Annual number of patients with invasive penile cancer and proportion receiving lymph node surgery over time, 2009–2020. No significantly increased temporal use of lymph node surgery was observed 2009–2014 (P = 0.053 from a univariate logistic regression with year as continuous covariate, restricted to the years before 2015). [file BCO2-7-e70220-s002.docx]

|  | Lymph node surgery, n (%) | Registrations, n |
| --- | --- | --- |
| Year of diagnosis |  |  |
| 2009 | 48 (51) | 95 |
| 2010 | 34 (60) | 57 |
| 2011 | 49 (58) | 84 |
| 2012 | 41 (57) | 72 |
| 2013 | 47 (69) | 68 |
| 2014 | 51 (62) | 82 |
| 2015 | 71 (78) | 91 |
| 2016 | 78 (74) | 105 |
| 2017 | 101 (76) | 133 |
| 2018 | 72 (78) | 92 |
| 2019 | 82 (77) | 106 |
| 2020 | 70 (74) | 94 |

Supplementary Table 1. Annual number of patients with invasive penile cancer and proportion receiving lymph node surgery over time, 2009-2020. No significantly increased temporal use of lymph node surgery was observed 2009-2014 (P=0.053 from a univariate logistic regression with year as continuous covariate, restricted to the years before 2015).
