# Supplementary material for: Lymph node surgery after centralization of penile cancer care in Sweden: Extent of use and complications
Source: BJUI Compass. 2026 Apr 30;7(5):e70220. doi: 10.1002/bco2.70220 (PMC13133551; doi:10.1002/bco2.70220)
Supplement: Supplementary file 2 — Figure S1. Distribution of complication rates (lymphedema/lymphocele/infection) after lymph node surgery before 2015 for Malmö/Örebro vs all other centers. [file BCO2-7-e70220-s001.pdf]

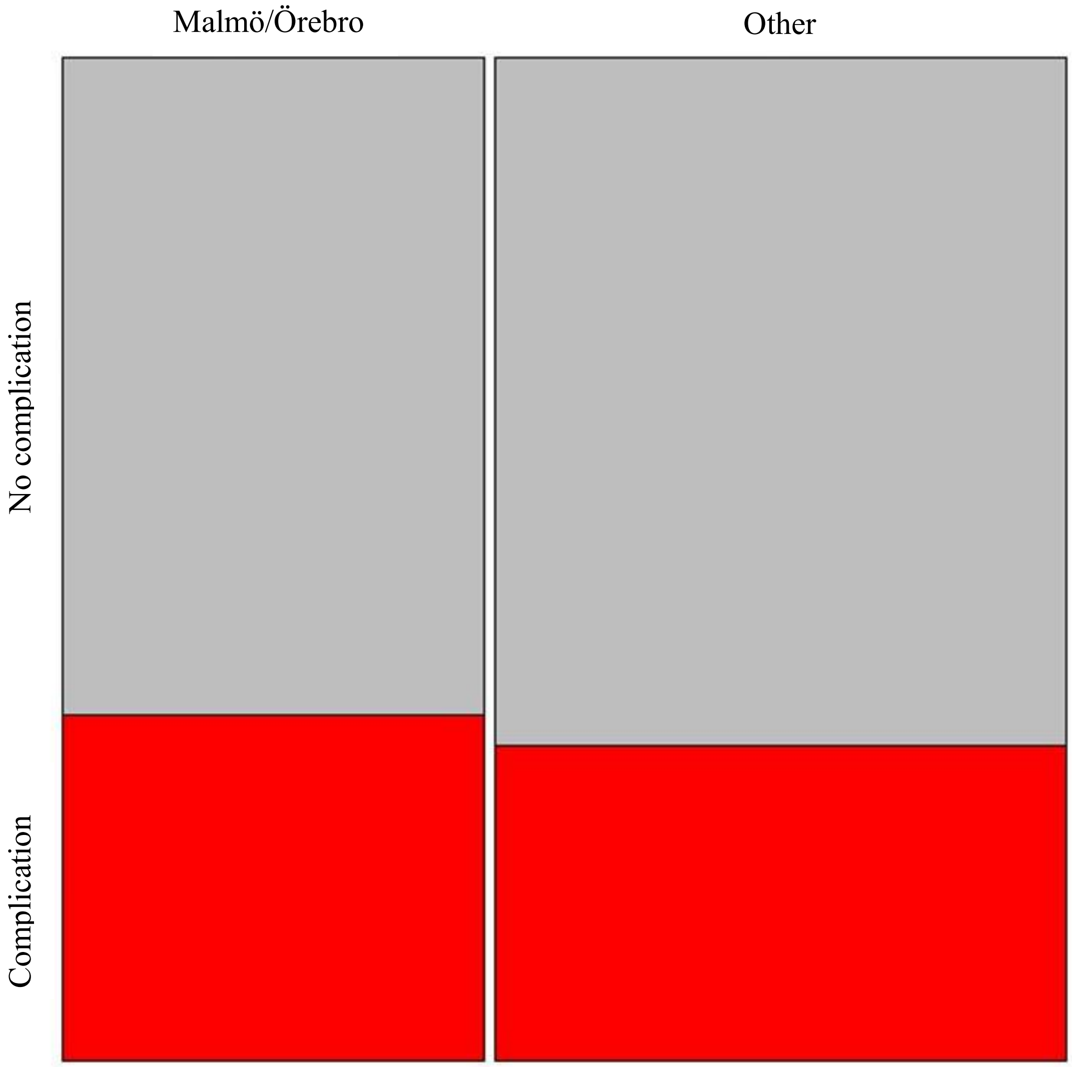

Supplementary Figure 1. Distribution of complication rates (lymphedema/lymphocele/infection) after lymph node surgery before 2015 for Malmö/Örebro vs all other centers.
